# Unilateral Cataract and Retinitis Pigmentosa in a Patient With Polyneuropathy, Hearing Loss, Ataxia, Retinitis Pigmentosa, and Cataract (PHARC) Syndrome: A Case Report

**DOI:** 10.7759/cureus.54295

**Published:** 2024-02-16

**Authors:** Miguel E Hernández-Emanuelli, Andres Emanuelli, Natalio Izquierdo

**Affiliations:** 1 School of Medicine, Ponce Health Sciences University, Ponce, PRI; 2 Ophthalmology, Medical Sciences Campus, University of Puerto Rico, San Juan, PRI; 3 Department of Surgery, School of Medicine, Medical Sciences Campus, University of Puerto Rico, San Juan, PRI

**Keywords:** retinitis pigmentosa, deaf-blindness, unilateral retinitis pigmentosa, cataracts, pharc syndrome

## Abstract

Patients with mutations in the α/β*-*hydrolase *(ABHD) 12* gene develop ocular complications including cataracts and retinitis pigmentosa (RP), as part of the polyneuropathy, hearing loss, ataxia, RP, and cataract (PHARC) syndrome. A chart review on a patient with a heterozygous mutation on the *ABHD12* gene underwent a comprehensive ophthalmic evaluation. Visual acuity was 0 and 1.3 (logMAR) on the right eye (OD) and left eye (OS), respectively. There was pseudophakia in the OS. Fundus examination in OD was normal and pale optic nerve, attenuated vessels, cystoid macular edema, and mid-peripheral bony spicules were found in OS. Visual field test showed a ring scotoma in the OS. Macular optical coherence tomography (OCT) and fundus autofluorescence were compatible with cystoid macular edema of the OS. The electroretinogram (ERG) of left eye was flat. Patient’s systemic findings included: polyneuropathy and hearing loss. Unilateral presentation of cataract and RP in a patient with a heterozygous pathogenic mutation on the *ABHD12* gene is rare. This could be due to mosaicism. Retinal follow-up is warranted in this patient since manifestations may occur later in the contralateral eye. A heterozygous pathogenic mutation on the *ABHD12 *gene may lead to partial ocular and systemic manifestations of the PHARC syndrome.

## Introduction

Fiskerstrand and colleagues first described patients with polyneuropathy, hearing loss, ataxia, retinitis pigmentosa (RP), and cataract (PHARC) syndrome, a rare neurodegenerative disorder [1,2]. PHARC syndrome affects the central and peripheral nervous systems [2-4]. It is characterized by sensorimotor polyneuropathy, hearing loss, cerebellar ataxia, RP, and early-onset cataracts. [2-4]. The PHARC syndrome forms part of one of the causes of hereditary deaf-blindness, leading to difficulty in accurate diagnosis [5]. Misdiagnosis is common among PHARC patients due to symptom similarity to other conditions such as Charcot-Marie Tooth disease, mitochondrial diseases, Refsum Disease, RP, and Usher syndrome [3,4].

PHARC syndrome is inherited as an autosomal recessive trait [6,7]. Pathogenic mutations in the *a/b-hydrolase 12 (ABHD12)* gene have been associated with PHARC syndrome [2-9]. The a/b-hydrolase domain-containing protein 12 (ABHD12), a hydrolytic enzyme related to endocannabinoid metabolism, is predominantly expressed in the central nervous system (CNS) and is encoded by the *ABHD12 *gene.

We report on the first patient with PHARC syndrome who developed unilateral cataract and RP.

This data was previously presented in part as a poster at the 2022 World Ophthalmology Congress on September 9-12, 2022.

## Case presentation

A 52-year-old patient was referred to our clinic due to vision and hearing loss. The patient reported decreased vision in the left eye (OS) and decreased hearing for over 15 years. Past medical history was remarkable for fibromyalgia, major depressive disorder, and sensorimotor disturbances, treated medically. The detailed medical history did not indicate any neurological issues in the parents and siblings. The patient underwent a comprehensive review of systems and ophthalmological evaluation. In addition, imaging and genetic studies were performed.

**Figure 1:**
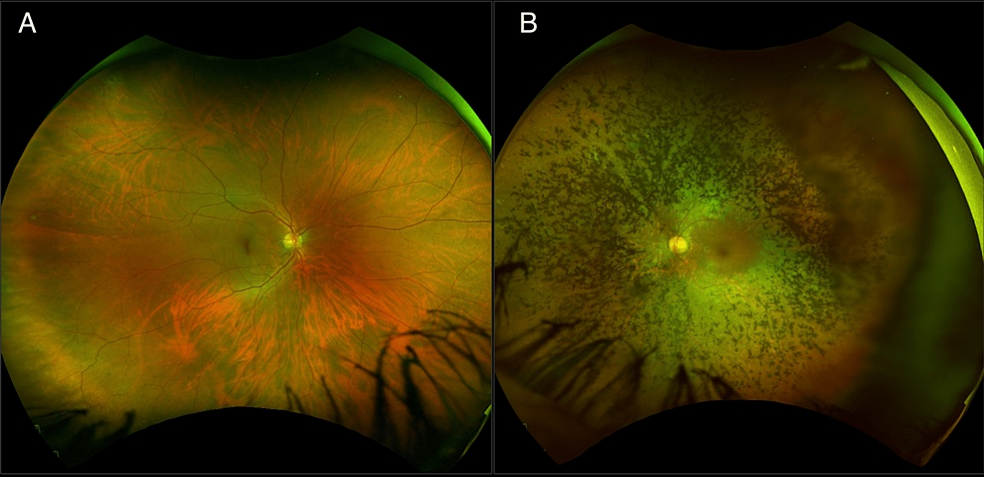
Color fundus photograph Color fundus photography depicts an intact macula and flat retina in the right eye. The left eye showed a pale optic nerve, attenuated vessels, cystoid macular edema, and mid-peripheral bony spicules in all quadrants.

Upon comprehensive ophthalmological evaluation, the patient has a best-corrected visual acuity of 20/20 (logMAR 0) and 20/400 (logMAR 1.3) on the right eye (OD) and left eye (OS), respectively. An electronic Snellen chart was used to assess the best-corrected visual acuity, which was then converted to LogMAR. Slit-lamp biomicroscopy revealed pseudophakia in the OS due to an ipsilateral early-onset cataract. As depicted in Figure 1A, the right eye was normal upon fundus examination. However, the patient had a pale optic nerve, attenuated vessels, cystoid macular edema, and mid-peripheral bony spicules on the left eye (Figure 1B).

**Figure 2:**
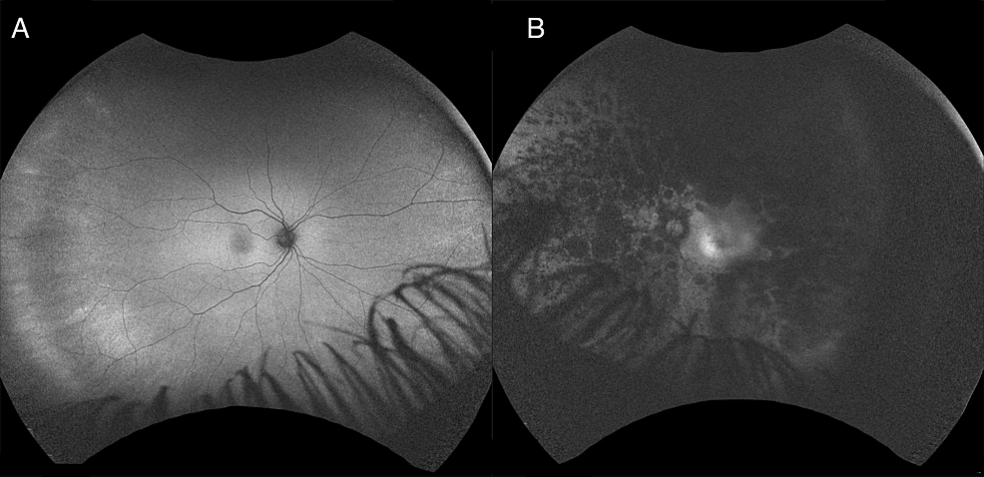
Fundus autofluorescence photograph Fundus autofluorescence shows a normal appearance of the right eye. There was an increased autofluorescent ring in the macula with granular patchy area of decreased autofluorescence in the left eye.

**Figure 3:**
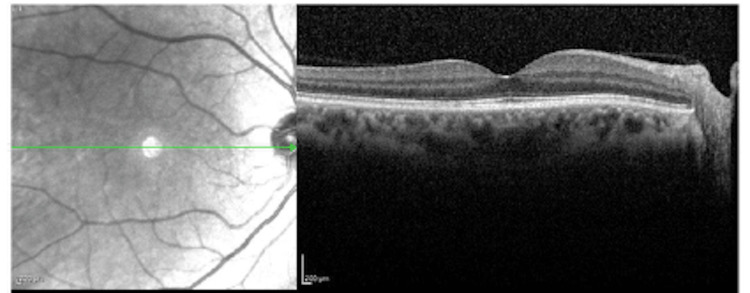
Macular OCT of the right eye Macular OCT of the right eye shows a normal foveal contour, intact inner and outer retinal layers and no evidence of intraretinal fluid. OCT: optical coherence tomography

**Figure 4:**
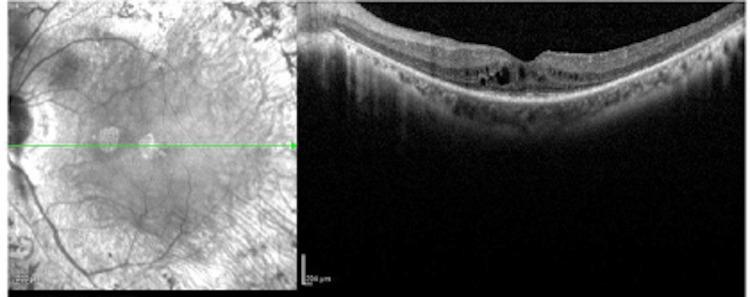
Macular OCT of the left eye Macular OCT of the left eye shows intraretinal fluid involving the central foveal subfield. Significant choroidal thinning was found in the macular area. Significant atrophy of the external retinal layers was found in temporal extrafoveal area. OCT: optical coherence tomography

**Figure 5:**
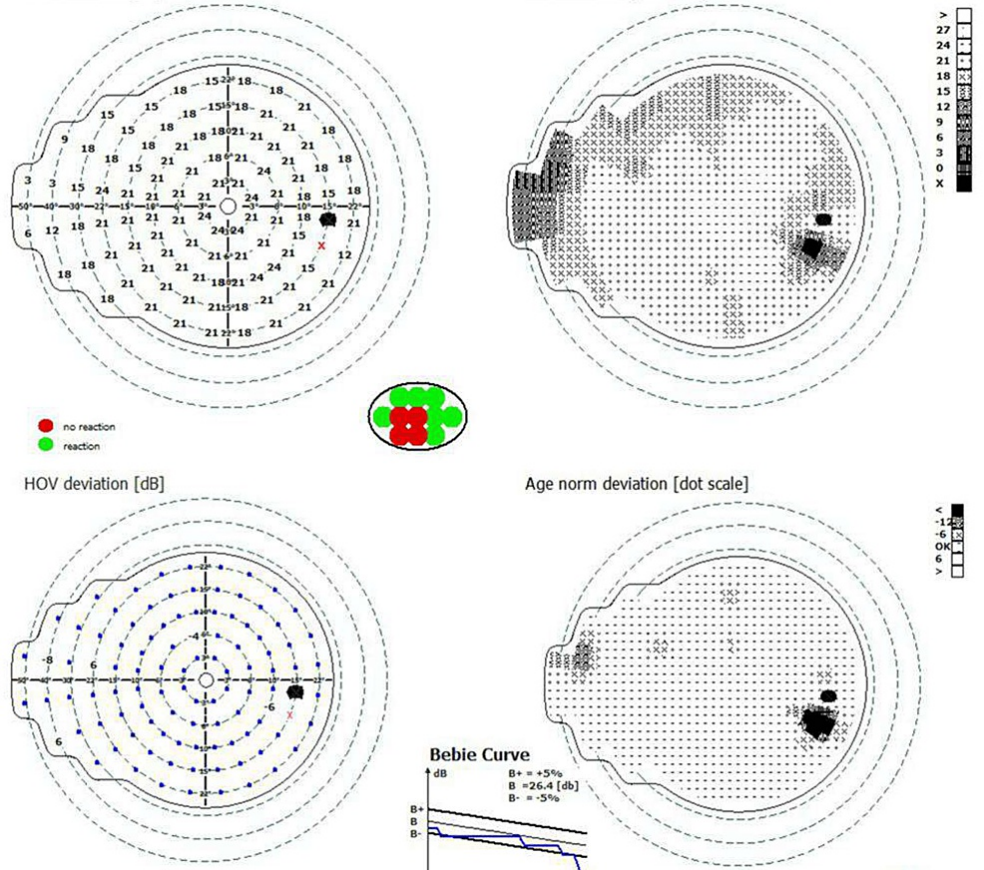
Visual field of the right eye Visual field result of the right eye revealed an intact visual sensitivity across the tested field, with no evidence of scotomas, defects, or abnormalities.

**Figure 6:**
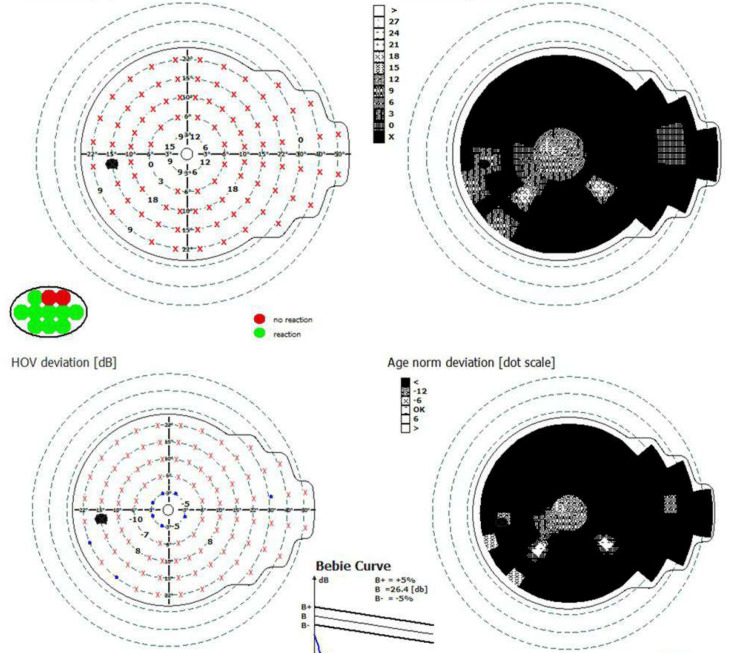
Visual field of the left eye Visual field result in the left eye shows a central island of vision.

Fundus autofluorescence (Figure 2B) and macular optical coherence tomography (OCT) (Figures 3-4) showed cystoid macular edema (CME) of the OS only. CME was treated with topical dorzolamide 2% three times a day (TID) and a follow-up examination revealed persistent intraretinal fluid with anatomical improvement. Upon Visual field test, the patient had a normal result in the OD (Figure 5) and a ring scotoma of the OS (Figure 6). Electroretinogram (ERG) results of the right eye were within normal limits and abolished in the left eye.

The audiometry test revealed bilateral mild sensorineural hearing loss. The magnetic resonance imaging (MRI) displayed non-specific mild white matter changes. Genetic studies showed a heterozygous pathogenic variant (deletion in exon 1) in the *ABHD12* gene (Table 1). Invitae Corporation’s genotyping microarray, employing next-generation sequencing (NGS), was used for mutational screening. Testing of 248 genes was performed using genomic DNA extracted from a blood sample using standard protocols. The Illumina technology is employed to sequence the targeted regions after enriching genomic DNA from the provided sample through a hybridization-based protocol.

**Table 1:** Invitae diagnostic testing results

Gene	Variant	Zygosity	Variant Classification
ABHD12	Deletion (Exon 1)	Heterozygous	Pathogenic
BBS9	Gain (Exons 1-2)	Copy number = 3	Uncertain significance
PRPF4	c.586G>A (p.Ala196Thr)	Heterozygous	Uncertain significance

## Discussion

To date, the literature encompasses 12 studies documenting a collective of 51 patients [9]. Patients with PHARC syndrome have multi-systemic manifestations, including polyneuropathy, ataxia, hearing loss, RP, and cataracts caused by mutations in the *ABHD12* gene [2-9]. The *ABHD12* gene, located on chromosome 20 and spanning 13 exons, encodes the αβ-hydrolase domain-containing protein 12. ABHD12 is a transmembrane protein with its active site situated at the extracellular surface [10]. Furthermore, ABHD12 exhibits enzymatic activities such as 2-arachidonoylglycerol (2-AG) hydrolysis and lysophosphatidylserine (LPS) lipase activity [11]. Studies involving *ABHD12* gene knock-out mice revealed an increase in the brain's LPS levels, accompanied by the development of a PHARC-related phenotype over time. This phenotype included impaired auditory function and motor behavior [11].

PHARC syndrome is a genetically heterogeneous and clinically variable disease, with full expression in early adulthood [9]. Nguyen and co-workers described that patients with PHARC syndrome have diverse clinical variability of onset, severity, and disease progression [4]. While the initial symptom noted in patients is hearing loss, neuropathy is the most prevalent clinical observation [9]. Sensorimotor polyneuropathy is observed in 91% of patients, with ataxia in 69%, hearing loss in 88%, RP in 78%, and cataracts in 86% [9].

Our patient had all of the above except for ataxia manifestations as part of the syndrome. These findings are compatible with the previous literature [2-9]. Previous studies reported that patients with the syndrome develop clinical findings simultaneously in both eyes [3-4]. Our patient had a heterozygous pathogenic mutation in the *ABHD12* gene, which led to unilateral ocular findings. Unilateral presentation of an early-onset cataract and RP in a patient with a heterozygous pathogenic mutation in the *ABHD12* gene is rare [3-4]. Patients with unilateral ocular findings may benefit from close follow-up since retinal manifestations may occur later in the contralateral eye. Limitations in our study include a parental segregation analysis.

It is important to note that all neurological, auditory, and ophthalmic manifestations may not be evident at the initial presentation [4,9]. Co-management of patients is warranted to address the multi-systemic manifestations of patients with the syndrome to maintain quality of life.

## Conclusions

A heterozygous pathogenic mutation in the *ABHD12* gene may lead to partial ocular and systemic manifestations as part of the PHARC syndrome. A multidisciplinary evaluation involving different specialists is recommended for patients with suspected or genetically confirmed PHARC syndrome due to the variability in symptoms and clinical findings. Genetic testing remains a valuable tool in confirming the diagnosis.

## References

[1] Fiskerstrand T, Knappskog P, Majewski J, Wanders RJ, Boman H, Bindoff LA (2009). A novel Refsum-like disorder that maps to chromosome 20. Neurology.

[2] Fiskerstrand T, H'mida-Ben Brahim D, Johansson S (2010). Mutations in ABHD12 cause the neurodegenerative disease PHARC: An inborn error of endocannabinoid metabolism. Am J Hum Genet.

[3] Dias Bastos PA, Mendonça M, Lampreia T, Magriço M, Oliveira J, Barbosa R (2021). PHARC syndrome, a rare genetic disorder—case report. Mov Disord Clin Pract.

[4] Nguyen XT, Almushattat H, Strubbe I (2021). The phenotypic spectrum of patients with PHARC syndrome due to variants in ABHD12: An ophthalmic perspective. Genes.

[5] Yoshimura H, Hashimoto T, Murata T, Fukushima K, Sugaya A, Nishio SY, Usami S (2015). Novel ABHD12 mutations in PHARC patients: the differential diagnosis of deaf-blindness. Ann Otol Rhinol Laryngol.

[6] Lerat J, Cintas P, Beauvais-Dzugan H, Magdelaine C, Sturtz F, Lia AS (2017). A complex homozygous mutation in ABHD12 responsible for PHARC syndrome discovered with NGS and review of the literature. J Peripher Nerv Syst.

[7] Frasquet M, Lupo V, Chumillas MJ, Vázquez-Costa JF, Espinós C, Sevilla T (2018). Phenotypical features of two patients diagnosed with PHARC syndrome and carriers of a new homozygous mutation in the ABHD12 gene. J Neurol Sci.

[8] Nishiguchi KM, Avila-Fernandez A, van Huet RA (2014). Exome sequencing extends the phenotypic spectrum for ABHD12 mutations: from syndromic to nonsyndromic retinal degeneration. Ophthalmology.

[9] Demir S, Sevik MO, Ersoy A, Geckinli BB, Sahin O, Arslan Ates E (2024). PHARC syndrome which an ultra-rare syndrome with retinitis pigmentosa and cataracts: Case report and review of the literature. Ophthalmic Genet.

[10] Blankman JL, Simon GM, Cravatt BF (2007). A comprehensive profile of brain enzymes that hydrolyze the endocannabinoid 2-arachidonoylglycerol. Chem Biol.

[11] Blankman JL, Long JZ, Trauger SA, Siuzdak G, Cravatt BF (2013). ABHD12 controls brain lysophosphatidylserine pathways that are deregulated in a murine model of the neurodegenerative disease PHARC. Proc Natl Acad Sci U S A.

